# P-2137. Evaluating the clinical utility of bronchoalveolar lavage polymerase chain reactions for the diagnosis of pulmonary infections in patients with hematological malignancies

**DOI:** 10.1093/ofid/ofae631.2292

**Published:** 2025-01-29

**Authors:** Rakan Al-Ghanamah, Katie Finnigan, Varshini Gali, Or Kalchiem-Dekel, Mini Kamboj, Tobias M Hohl, Esther Babady, Genovefa Papanicolaou, Yeon Joo Lee

**Affiliations:** Memorial Sloan Kettering Cancer Center, New York, New York; Memorial Sloan Kettering Cancer Center, New York, New York; Weill Cornell Medicine, New York, New York; Memorial Sloan Kettering Cancer Center, New York, New York; MSKCC, New York, NY; Memorial Sloan Kettering, New York, New York; Memorial Sloan Kettering, New York, New York; Memorial Sloan Kettering Cancer Center, New York, New York; Memorial Sloan Kettering Cancer Center, New York, New York

## Abstract

**Background:**

Invasive pulmonary infections are a significant cause of morbidity and mortality in patients with hematological malignancies and hematopoietic stem cell transplantation (HCT) recipients. A delay in identifying a causative agent may result in late initiation of appropriate treatment and worse outcomes. We explore the diagnostic utility of incorporating commercially available polymerase chain reaction (PCR)-based assays performed on bronchoalveolar lavage (BAL) specimens into evaluating invasive pulmonary infections in patients with hematological malignancies and HCT recipients.

**Figure:**
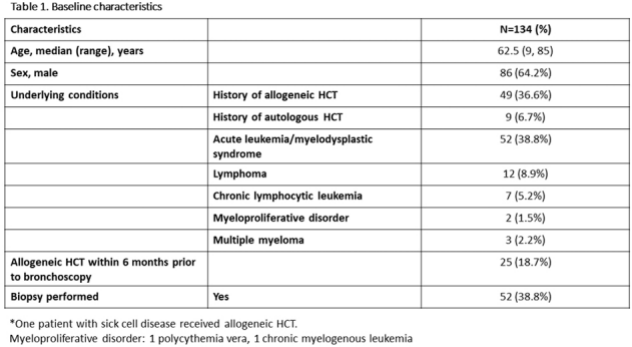

**Methods:**

Patients with hematological malignancies and HCT recipients who underwent a bronchoscopy 1/2020 - 1/2024 with at least one PCR assay (Aspergillus, Mucorales, and Nocardia) sent to Viracor (Lenexa, KS) were included. Multiple bronchoscopies were captured if performed ≥6 weeks apart. “Proven” and “Probable” invasive fungal infection (IFI) were defined as per EORTC/MSG 2020 guidelines. Pulmonary infection was defined by clinical findings, galactomannan antigen (GMA) testing, culture from BAL, histopathology if a biopsy was performed, and radiology imaging studies. Sensitivity, specificity, positive predictive value (PPV), and negative predictive value (NPV) of PCRs and BAL GMA were tested for probable and proven IFI and pulmonary nocardia infection.

**Figure:**
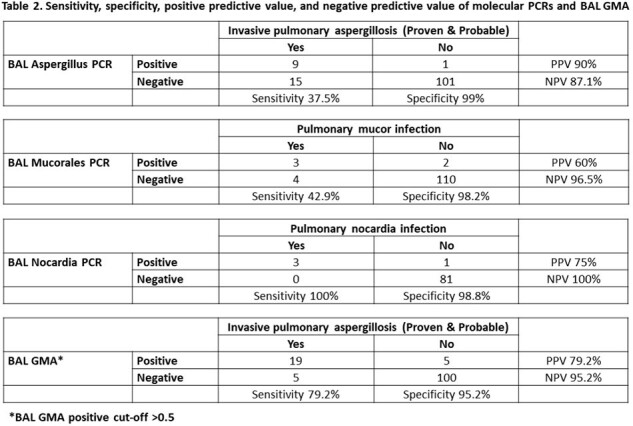

**Results:**

Figure 1 describes the study cohort. Fifty-eight of 134 (43.3%) received HCT (Table 1). The sensitivity, specificity, PPV, and NPV for BAL PCRs, and BAL GMA are provided in Table 2. Figure 2 shows diagnosis by standard of care and BAL PCRs. Among 19 patients with positive BAL GMA, 4 were positive with culture. Two patients were negative for BAL GMA and BAL Aspergillus PCR, but culture was positive for *A. fumigatus*. Culture was negative for all pulmonary mucor, and culture was positive for all pulmonary nocardia.

**Figure:**
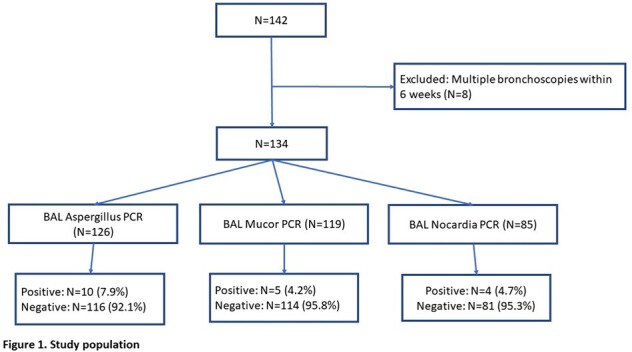

**Conclusion:**

Aspergillus PCR has lower sensitivity & NPV, and higher specificity & PPV compared to BAL GMA. The sensitivity of Mucorales and Nocardia PCRs was 42.9% and 100%, respectively. The specificity of Mucorales and Nocardia PCRs were 98.2% and 98.8%, respectively. Our study suggests PCRs from BAL can be helpful as an adjunct tool in diagnosing invasive pulmonary infections in highly immunocompromised patients.

**Figure:**
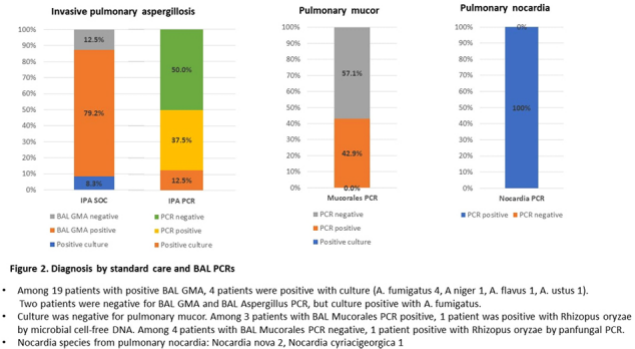

**Disclosures:**

Genovefa Papanicolaou, MD, AlloVir: Advisor/Consultant|AlloVir: Data safety monitoring committee|Merck: Advisor/Consultant|Merck: Grant/Research Support|Merck: Investigator|Symbio: Advisor/Consultant Yeon Joo Lee, MD, MPH, AiCuris: institutional research support for clinical trials|Karius: institutional research support for clinical trials|Merck: Grant/Research Support|Scynexis: institutional research support for clinical trials

